# Studies on reproductive strategies of *Vitex negundo* L. var. *heterophylla* (Franch.) Rehder (Lamiaceae) based on morphological characteristics and SSR markers

**DOI:** 10.1002/ece3.6271

**Published:** 2020-04-22

**Authors:** Xiaohan Sun, Feng Wang, Rong Cui, Xiao Liu, Xiangxiang Li, Jibin Dong, Lu Sun, Siqi Qin, Renqing Wang, Peiming Zheng, Hui Wang

**Affiliations:** ^1^ Institute of Ecology and Biodiversity School of Life Sciences Shandong University Qingdao China; ^2^ Shandong Provincial Engineering and Technology Research Center for Vegetation Ecology Shandong University Qingdao China; ^3^ Qingdao Forest Ecology Research Station of National Forestry and Grassland Administration Shandong University Qingdao China

**Keywords:** mating system, morphological characteristics, reproductive strategies, SSR markers, *Vitex negundo* var. *heterophylla*

## Abstract

*Vitex negundo* L. var. *heterophylla* (Franch.) Rehder (Lamiaceae) is an important tree species for soil and water conservation, yet the reproductive ecology of this species remains to be elucidated. To investigate the reproductive traits of *V. negundo* var. *heterophylla*, the phenology, morphological characteristics (a suite of characters was assessed: floral morphology, nectar production, pollen viability, and stigma receptivity) and mating system of this species were systematically revealed for the first time in this study. Phenological observations, morphological measurements, and nectar production analysis were conducted during anthesis. Pollen viability and stigma receptivity at different flowering stages were measured by biochemical methods. Finally, genetic analysis based on SSR markers was used to reveal the mating system; outcrossing index and pollen‐ovule ratio were also calculated to help analysis. *V. negundo* var. *heterophylla* showed several obvious characteristics of outcrossing, such as abundant and attractive flowers, secreting nectar, and emitting scent. In addition, mechanisms such as homogamy and a short anther‐stigma distance that can promote self‐fertilization were also identified in this species. The coexistence of selfing and outcrossing characteristics demonstrates a predominantly outcrossed mixed mating system (outcrossing rate, *t* = 95%). The scientific information provided by this study may contribute to conservation of *V. negundo* var. *heterophylla* from a reproductive perspective.

## Graphical abstract

To investigate the reproductive traits of *Vitex negundo* var. *heterophylla *and propose conservation strategies, the phenology, morphological characteristics, and mating system of this species were systematically revealed for the first time in this study. *V. negundo* var. *heterophylla *presents a predominantly outcrossed mixed mating system with self‐compatibility and inbreeding depression at the seedling emergence stage. Conservation strategies such as selective thinning and effective in‐suit protection should be carried out to ensure the stability of the population.

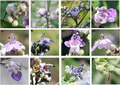

## 1 INTRODUCTION

Knowledge about plant reproductive traits is critical for determining the barriers to propagule production and developing comprehensive conservation plans (Montagna, Silva, Pikart, & Reis, 2018; Nazareno & Reis, 2012; Sinébou, Quinet, Ahohuendo, & Jacquemart, 2016). Flowering phenology, floral morphological characteristics, and mating systems are key factors of plant reproductive biology (Pool‐Chalé, Ramírez‐Morillo, Fernández‐Concha, & Hornung‐Leoni, 2018; Sá, Furtado, Ferrero, Pérezarrales, & Consolaro, 2016), and all share a mutual connection.

Flowering phenology (the periodicity of flower production) follows a specific schedule ranging from complete synchrony within the population to full asynchrony. While flowering asynchrony between plants may cause temporal isolation, flowering synchrony may shape mating possibilities between individuals by influencing pollination patterns (Ison, Stuart, Diedre, & Ashley, 2014). Floral morphological characteristics (the form and function of sexual units) involve areas of study such as floral display traits, nectar production rewards, stigma receptivity, and pollen viability. The variation in floral traits is strongly associated with evolution of mating systems (Goodwillie et al., 2010; Schoen, 1982). Moreover, the morphology and function of stamens and pistils provide information about reproductive features including (a) gender and degree of dichogamy, creating different probabilities of pollen transfer at different positions and thus influencing sex allocation (Brunet & Charlesworth, 1995); (b) anther‐stigma distance (ASD), affecting the degree of self‐pollen deposition on stigmas and thus therefore the outcrossing strategy (Chang & Rausher, 1998); and (c) pollen‐ovule ratio (P/O), an integral part of a plant's mating system (Cruden, 1977). These traits have a significant impact on reproductive fitness directly or indirectly.

In plants, the mating system (whether obligate outcrossing, autonomous selfing, or mixed mating) can have a profound effect on the genetic variation of a species and the genetic composition of natural populations. Traditionally, plant reproductive traits are inferred from phenology, morphological characteristics, and manipulative pollination experiments (Jorge, Loureiro, & Castro, 2015; Murren et al., 2014; Pool‐Chalé et al., 2018). However, these observations specifically measure the relative degree of outcrossing and selfing, and the true outcrossing rate of natural populations cannot be reflected. Such limitations can be overcome using genetic markers. Microsatellite markers (SSR) are widely used in various genetic analyses as powerful molecular marker tools due to their high efficiency and polymorphism (Coelho et al., 2018; Collevatti, Grattapaglia, & Hay, 2010; Sharma, Islam, Negi, & Tripathi, 2017). The mating system parameters estimated from genetic markers contribute to the development of conservation strategies for a given species.

The genus *Vitex* (Lamiaceae) includes approximately 300 species, mainly occurring in the tropics (Kok, 2007). *Vitex negundo* L. var. *heterophylla* (Franch.) Rehder (Lamiaceae) is a dominant perennial deciduous shrub native to China. It is drought‐tolerant and a typical pioneer species that is widespread on hillsides, gorges, or rocky gaps in northern China (Du, Guo, Zhang, & Wang, 2010). Because of its important medical value, studies involving the *Vitex* species focus primarily on phytoconstituents, biological activities, and clinical potential (Rani & Sharma, 2013; Sena et al., 2008; Yao et al., 2016), and few reproductive studies have been conducted. Furthermore, there are no systematic or comprehensive studies on the reproductive ecology of *V. negundo* var. *heterophylla*.

Soil erosion has become one of the most serious global environmental problems. Since the 1950s, the Chinese government has implemented vegetation rehabilitation as a critical measure of soil and water conservation (Zhao, Mu, Wen, Wang, & Gao, 2013). During the process of slope vegetation recovery, the growth of shrub is often slowed or halted entirely due to the excessive growth of herb plants in the early stage of planting and the fierce competition between herb and shrub (Liao, Agen, Xu, & Gu, 2006; Tao, Jiang, Wei, & Tian, 2011). In addition, vegetation is highly vulnerable to the effects of human activities, such as grazing and forest management practices (Erb et al., 2018). Consequently, it is difficult to form stable communities with native plants as dominant species, such as *V. negundo* var. *heterophylla*, during vegetation restoration. It is therefore essential to understand the reproductive ecology of certain species to develop effective strategies for its conservation and management. Hence, we investigated reproductive traits of *V. negundo* var. *heterophylla* including (a) flowering phenology; (b) morphological characteristics: floral morphology, nectar rewards, stigma receptivity, and pollen viability; and (c) mating system based on SSR markers, outcrossing index (OCI), and P/O. The findings presented here will shed light on research gap in the current literature and contribute to the development of successful vegetation restoration strategies.

## 2 MATERIALS AND METHODS

### 2.1 Study site

The studies presented here were conducted in a natural population (30 × 30 m with a density of 5.3 individuals per 100 m^2^) at Fanggan Research Station of Shandong University, Shandong Province, China (36°26′N, 117°27′E). The study site belongs to the warm temperate monsoon continental climate, with an annual average temperature of 13 ± 1°C, and an annual average precipitation of 600–830 mm, most of which occurs in July and August (Du et al., 2010). According to our measurements, the content of total levels of nitrogen and air‐dried moisture in soil during the summer were 0.22 ± 0.02% and 2.58 ± 0.24%, respectively (*M* ± *SE*). In our study sites, *V. negundo* var. *heterophylla* was identified as the dominant species (the ground diameter ranging from 1 to 20 cm) and *Ziziphus jujuba* var. *spinosa* (Rhamnaceae) as the subdominant species.

### 2.2 Flowering phenology

From March to December 2018, the phenology of 10 individuals (ground diameter >10 cm, growing evenly, approximately 10 m apart) of *V. negundo* var. *heterophylla* at the study area was observed, and the germination, leaf‐expansion, flowering, fruiting, and deciduous stages were recorded. Moreover, 5 inflorescences × 10 individuals (*n* = 50, in total) were marked with durable hang tags from the bud phase. Buds were observed every 2 days until the flowers opened. The observations were made every day during anthesis to assess the flowering habits of inflorescences and single flowers. The marked inflorescences were monitored weekly during the fruiting phase, and the time of fruit expansion and ripening was recorded.

### 2.3 Morphological characteristics

#### 2.3.1 Floral morphology

We observed the type, sex, color, and structure of androecium and gynoecium of open flowers on the same 10 individuals, and the spatial position of stigma and anthers were recorded. Moreover, various morphological observations were performed every 2 hr after flowering to record the functional floral morphology. These measurements included petal development, anther dehiscence process, the order and time of flower organ wilting, odor release, and nectar secretion. Moreover, 50 flowers (5 flowers × 10 individuals, *n* = 50, in total) were randomly selected at the same flowering time (petals were fully expanded without withering) for the measurement of floral parameters using caliper (Table 2).

#### 2.3.2 Nectar rewards

Flowers were bagged with paper pollination bags to exclude pollinators for 6 hr (5 flowers × 5 individuals, *n* = 25, in total) or 24 hr (5 flowers × 5 individuals, *n* = 25, in total) before evaluation. Nectar volume per flower was evaluated at 6 and 24 hr after anthesis using a 1‐μl microcapillary tube. Subsequently, sugar concentration was measured with a portable hand refractometer and expressed as the percentage of sucrose. According to the formula in Sinébou et al. (2016), the sugar concentration (%) was transformed into mg/μl, which remained unchanged during flowering. The total sugar content of nectar per flower (mg) was obtained by multiplying the nectar volume (μl) by the sugar concentration (mg/μl). A *t* test was employed to compare nectar volume at 6 and 24 hr after anthesis. The statistical analysis and plot were performed using R 3.4.3 software (R Development Core Team, 2017), with a significance threshold set at .05.

#### 2.3.3 Stigma receptivity

Stigma receptivity was measured via H_2_O_2_ test (Dafni, 1992) at 0, 3, 6, 9, and 12 hr after anthesis. Ten stigmas were randomly collected from three individuals at each stage and immediately immersed in a plastic bottle cap containing hydrogen peroxide (3% concentration) reaction solution. The presence and number of bubbles around stigmas indicating receptivity were observed under a magnifying glass (11×). Stigma morphology of each stage was also recorded at the same time.

#### 2.3.4 Pollen viability

Pollen viability was determined by 2,3,5‐triphenyl tetrazolium chloride test (TTC) (Dafni, 1992) at 0, 3, 6, 9, 12, and 24 hr after anthesis. Ten anthers of different flowers were randomly collected from 3 individuals at each stage, and their pollen grains were placed on clean microscope slides and mixed, and a drop of TTC (0.5%) solution was added immediately. Each slide was then placed in a Petri dish with wet filter paper and incubated for 2 hr at 37°C in dark environment. Viable pollen grains were stained red, whereas inviable grains remained unstained. The number of stained and total pollen grains was quantified in five random visual fields on each slide using an optical microscope (CX31RTSF; Olympus), and the proportion of red grains was used to evaluate pollen viability.

### 2.4 Mating system

#### 2.4.1 SSR based analysis of the reproductive traits

In October 2018, five mother trees were selected during the fruiting season. From each mother tree, expanded leaves were collected and stored in plastic bags containing silica gel, and seeds were brought back to the greenhouse to raise seedlings. All progeny leaves were harvested in January 2019, and five families of open‐pollinated progeny arrays with a total of 90 individuals (85 progenies and 5 seed parents) were genotyped. The number of genotyped progenies ranged from 8 to 26 per seed tree.

Genomic DNA extraction from dried leaf tissue was performed followed the CTAB method (Doyle & Doyle, 1987). We used nine polymorphic loci (V02, V07, V25, V49, V55, V76, V95, V97, and V100) developed by Liu et al. (2019) for microsatellite analysis. The system and procedure of PCR amplifications were carried out using the same criteria as described in Liu et al. (2019). Product sizes were measured using an ABI 3730xl DNA capillary sequencer (Applied Biosystems) with a LIZ 500 Internal Size standard (Applied Biosystems) and Peak Scanner software version 1.0 (Applied Biosystems). The presence of one of the maternal alleles in each progeny individual was used to confirm the inheritance of microsatellite marker loci.

In the case of small family sizes and known maternal genotype information, the analysis of mating systems is based on a Bayesian method, using the program BORICE developed by (Koelling, Monnahan, & Kelly, 2012) suitable for the co‐dominant SSR marker. We estimated mating parameters including the population outcrossing rate (*t*), inbreeding coefficient (*F*), and maternal individual inbreeding histories (IH value = *C_k_*: number of generations of selfing in the ancestry of individual *k*). Before running BORICE, we set up a chain of 100,000 steps with a burn‐in of the first 10,000 steps and allowed the existence of null alleles at all marker loci.

#### 2.4.2 OCI and P/O

The OCI was developed by Dafni (1992) and calculated at the species level based on morphological characteristics which were observed and measured to predict the mating system. The OCI assignment method is given in Table 1, and the value of OCI is obtained by adding the scores of each judgment condition. The inflorescence diameter, flower size, and flowering behavior were measured, and the OCI was evaluated according to Dafni (1992). For calculating the P/O (Cruden, 1977), a total of 150 mature but indehiscent anthers were collected from three individuals (50 for each) and placed in three centrifuge tubes respectively containing 10 ml 0.1% hydrochloric acid solution. Pollen grains in each tube were shaken up after water bath for 4 hr at 55°C; then, the suspension was removed onto a slide immediately, and the number of grains was counted. P/O was determined by the ratio of the number of pollen grains per flower (the number of grains per anther multiplied by the number of anthers) to the number of ovules.

**TABLE 1:** Outcrossing index assignment method

Judgment condition	Score			
	0	1	2	3
Diameter of the flower or the inflorescence	<1 mm	1–2 mm	2–6 mm	>6 mm
Temporal separation of anther dehiscence and stigma receptivity	Hermaphrodite or protogyny	Protandry		
Spatial positioning of stigma to anthers	Same height	Spatially separated		

## 3 RESULTS

### 3.1 Flowering phenology

The germination and leaf‐expansion stage occurred during March–May, and flower buds appeared at the beginning of June. Flowering lasted 3 months (June–August) and reached a peak by mid‐July. Flowering habits of inflorescences indicated that the inflorescence was panicle, which produced 2–20 flowers a day. The blossom order of an inflorescence was from the bottom to the top, and approximately 20 days were required for all flowers to open. Although flowering asynchrony existed among individuals, flowers within an inflorescence displayed a significant degree of synchrony. The blooming time was from approximately 9:00 to 11:00. The duration of a single flower was roughly 1–2 days according to our observation, and this period could be extended in rainy and cloudy days. Successfully fertilized flowers started fruit development immediately, and the time required to produce mature fruits was roughly 30–45 days. Thus, the fruiting period occurred between July and September, whereas the deciduous period began in October and lasted until March next year.

### 3.2 Morphological characteristics

#### 3.2.1 Floral morphology

**FIGURE 1:**
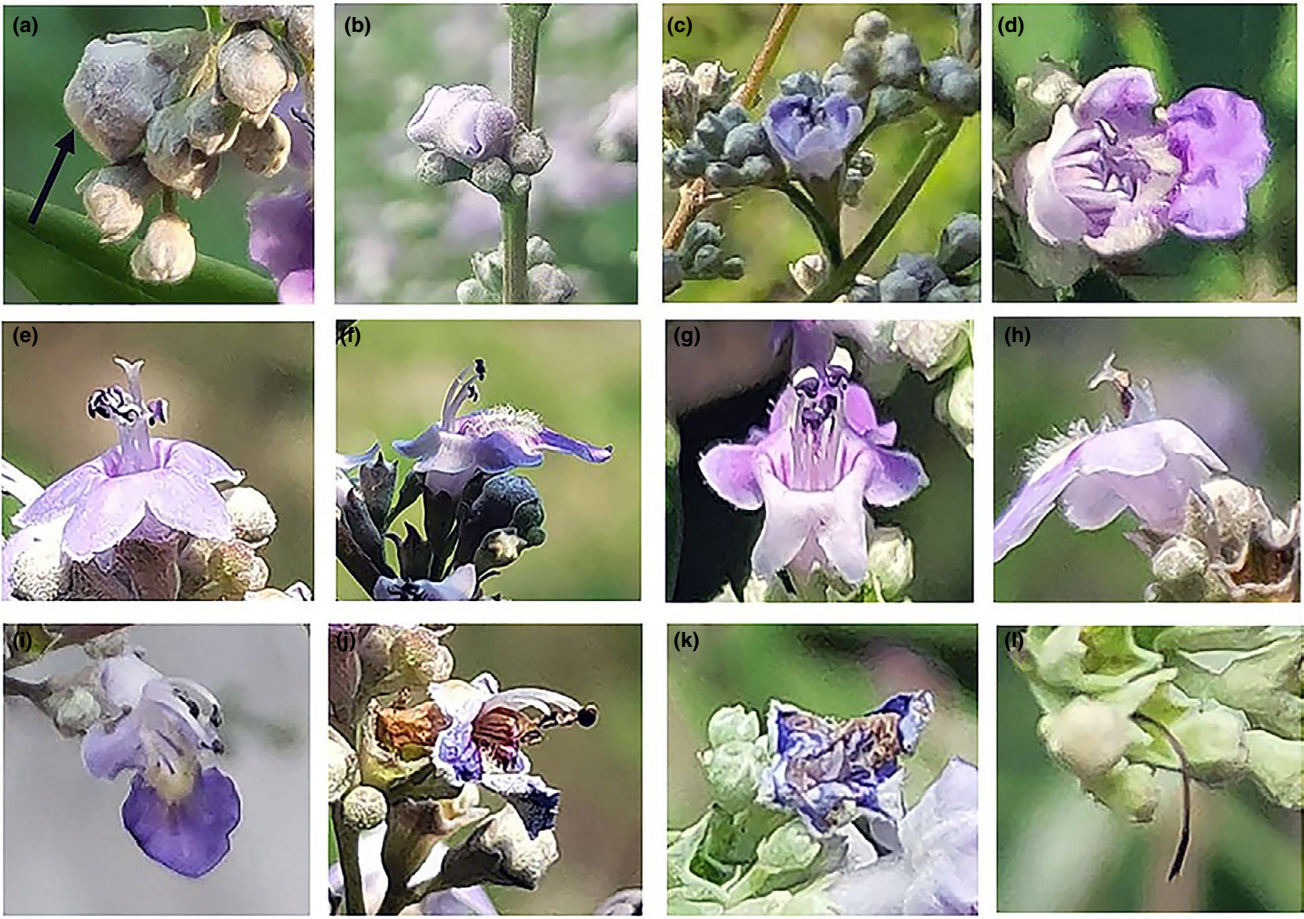
Flowering dynamics of *Vitex negundo* var. *heterophylla*. (a) Mature bud; (b–f) petals gradually unfolded with anthers dehisced; (g) anthers exposed large amounts of white pollen grains; (h) the stigma stretched out its lobes gradually; (i–l) the corolla and stamens withered gradually and fell off together, the style and stigma dried up and drooped, and fell off soon

**FIGURE 2:**
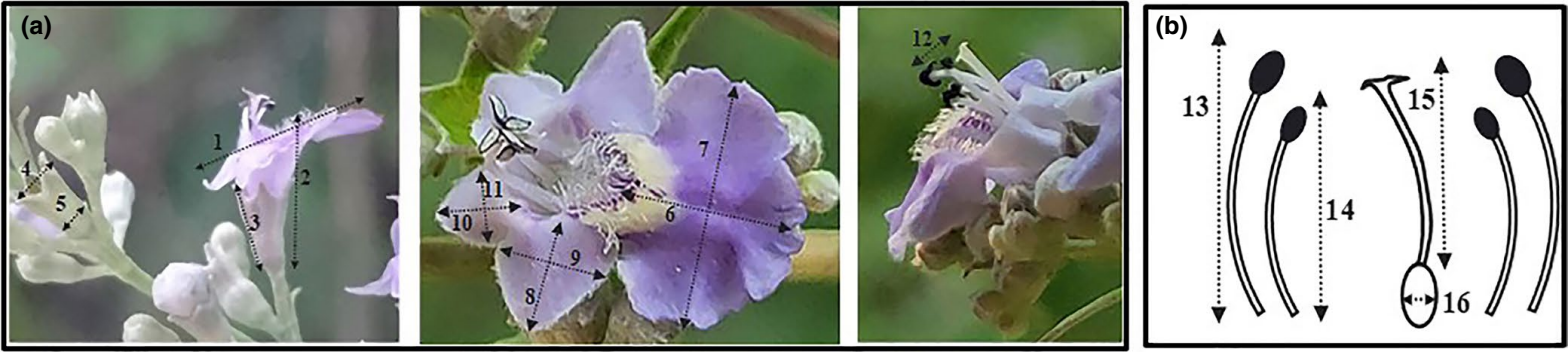
Measured floral parameters of *Vitex negundo* var. *heterophylla*. (a) Flower traits; (b) androecium and gynoecium traits. 1: corolla diameter, 2: corolla length, 3: sepal length, 4: sepal diameter, 5: sepal base diameter, 6: lower petal length, 7: lower petal width, 8: lateral fused petal length, 9: lateral fused petal width, 10: small fused petal width, 11: small fused petal length, 12: anther‐stigma distance, 13: long stamen length, 14: short stamen length, 15: style length, 16: ovary width

Flowers were hermaphroditic, zygomorphic purple, and possessed a floral fragrance. The bilabiate corolla was tubular at the base and composed of two fused petals in the upper lip and three in the lower lip (Figure 1). The corolla tube was covered by dense hairs. Four exserted stamens were situated adjacent to upper corolla lip and were didynamous. Anthers were dithecous, purple, and contained white pollen (Figure 2a). The ovary was superior, globose with four locules, each locule was 1‐ovuled. The bifid stigma was located between the long and short stamens (Figure 1f), or higher than long stamens (Figure 1e), indicating that flowers were partially herkogamous. From the measurement results, the change of stamen length was higher than that of style, and the length of long stamen displayed the highest level of fluctuation (Table 2). Table 2 outlines the floral parameters in detail.

**TABLE 2:** Descriptive statistics of the floral attributes of *Vitex negundo* var. *heterophylla* in the studies site (*n* = 50)

Floral attributes	Average	*SD*	Maximum	Minimum
Corolla diameter (mm)	6.77	0.39	7.59	6.05
Corolla length (mm)	4.11	0.33	4.83	3.46
Sepal length (mm)	2.87	0.33	3.70	2.24
Sepal diameter (mm)	2.08	0.16	2.46	1.84
Sepal base diameter (mm)	1.24	0.18	1.55	0.89
Lower petal length (mm)	4.94	0.61	6.50	4.30
Lower petal width (mm)	4.23	0.84	5.80	2.80
Lateral fused petal length (mm)	2.31	0.27	2.70	1.60
Lateral fused petal width (mm)	2.06	0.31	2.80	1.60
Small fused petal width (mm)	1.95	0.29	2.30	1.30
Small fused petal length (mm)	2.48	0.26	3.00	2.10
Anther‐stigma distance (mm)	0.59	0.20	0.90	0.20
Long stamen length (mm)	5.27	0.62	6.48	4.11
Short stamen length (mm)	4.38	0.43	5.22	3.80
Style length (mm)	5.19	0.37	5.83	4.42
Ovary width (mm)	0.88	0.14	1.10	0.70

The flowering dynamic of a flower can be divided into the following stages: (a) Bud stage (Figure 1a): tender buds that develop gradually; (b) Corolla elongation stage (Figure 1b): well‐developed perianth, flowers open once floral visitors touch any petal; (c) Initial blooming stage (Figure 1c–d): petals gradually extended to fully open state, the stamens, style and stigma exposed beyond the corolla tube, the anthers dehisced along the longitudinal splits; (d) Full blooming stage (Figure 1e–h): shortly after flowering, the color of anthers change from light purple to deep purple with a large amount of white pollen released, and the stigma gradually stretched out its lobes simultaneously; (e) Withering stage (Figure 1i–l): the outer edge of petals begin to curl and wither, stamens with dry anthers gradually curved toward the broad lobe of the lower lip and fall off with the corolla 24 hr after flowering. At this time, the style and stigma withered and drooped, developed a black‐brown color, and fell off by the afternoon of the second day. The calyx was persistent and partially enclosed the growing fruit.

#### 3.2.2 Nectar rewards

**FIGURE 3:**
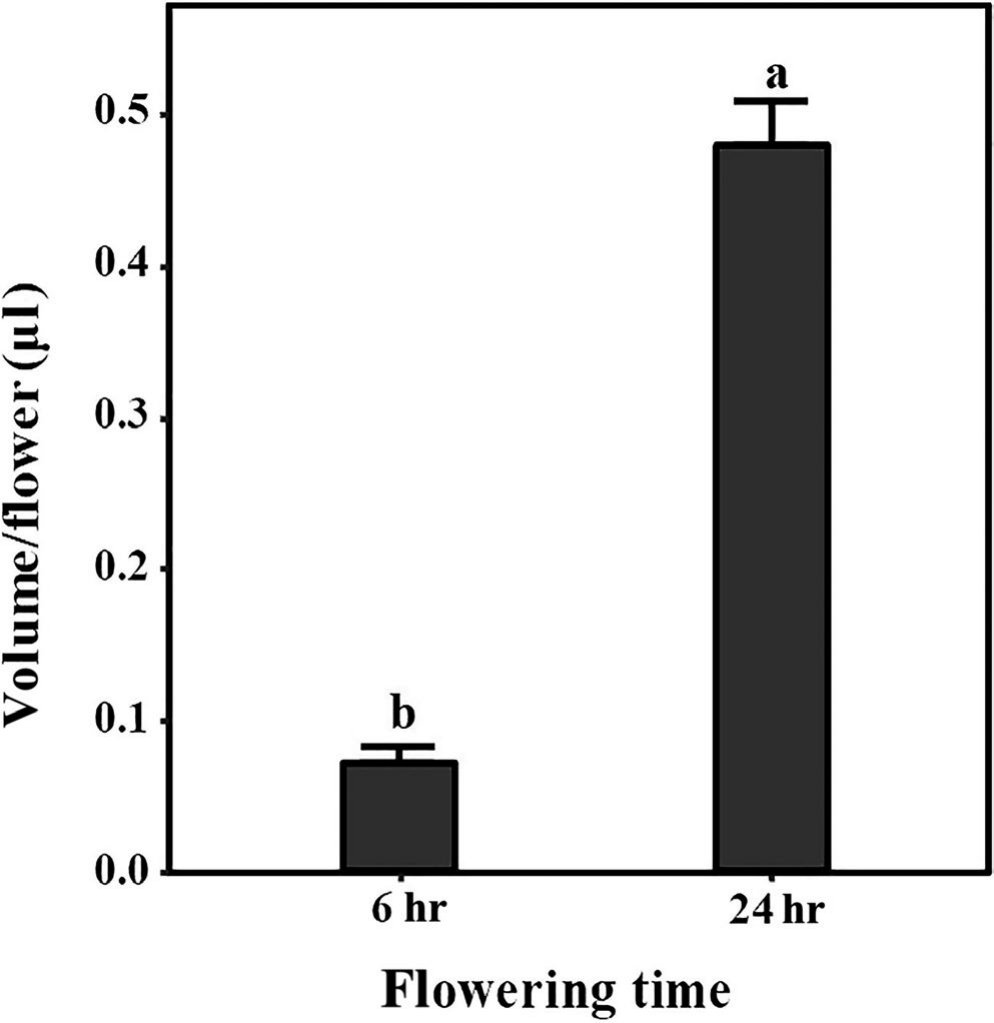
Nectar volume produced within 6 and 24 hr after anthesis per flower. Data are shown as *M*s + *SE*. Different letters represent significant difference at *p* < .05

These flowers produced small amounts of concentrated nectar at the base of corolla tube, mainly within 24 hr of anthesis. According to our observations, a flower secreted 0.073 ± 0.008 μl of nectar in 6 hr of anthesis. During the entire flowering period, a total of 0.478 ± 0.028 μl of nectar was secreted per flower, which was approximately six times higher than the amount of nectar produced at the beginning of flowering (Figure 3). The sugar concentration in nectar varied from 33% to 58% and averaged to 46.6 ± 8.60%, we used the average value of 0.56 mg/μl after unit conversion for later calculation (Sinébou et al., 2016). The total sugar content in nectar of a flower within 6 and 24 hr of anthesis was 0.041 ± 0.005 mg and 0.268 ± 0.016 mg, respectively, which followed the same trend as that of nectar secretion.

#### 3.2.3 Stigma receptivity and pollen viability

**FIGURE 4:**
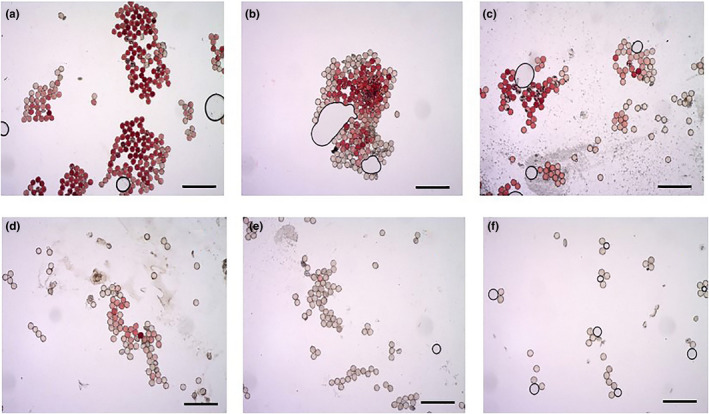
Pollen viability observations of *Vitex negundo* var. *heterophylla* at (a) 0, (b) 3, (c) 6, (d) 9, (e) 12, (f) 24 hr after anthesis. Only one representative picture is shown in each period. Scale bars = 100 μm

Stigma remained receptive during the whole flowering period and reached its peak within 3–6 hr of flowering. After 9 hr of flowering, stigma drooped and gradually lost receptivity (Table 3). Pollen viability reached its maximum at the beginning of flowering (81.76%, Figure 4a) and then gradually decreased, indicating homogamous flowers. Pollen viability was 71.68% (Figure 4b), 64.85% (Figure 4c), 24.72% (Figure 4d), and 16.8% (Figure 4e) after 3, 6, 9, and 12 hr of anthesis, respectively (Table 3). After 24 hr of anthesis, pollen viability was almost completely lost, with only 0.59% remaining (Figure 4f).

**TABLE 3:** Stigma receptivity and pollen viability of *Vitex negundo* var. *heterophylla* (*n* = 10)

Time after anthesis (hr)	Stigma receptivity	Pollen viability (%)
0	++	81.76
3	+++	71.68
6	+++	64.85
9	++	24.72
12	+	16.80
24	−	0.59

Note

−, means no stigma receptivity; +, means stigmas have low receptivity; ++, means stigmas have a medium level of receptivity; +++, means stigmas have high receptivity.

### 3.3 Mating system

**FIGURE 5:**
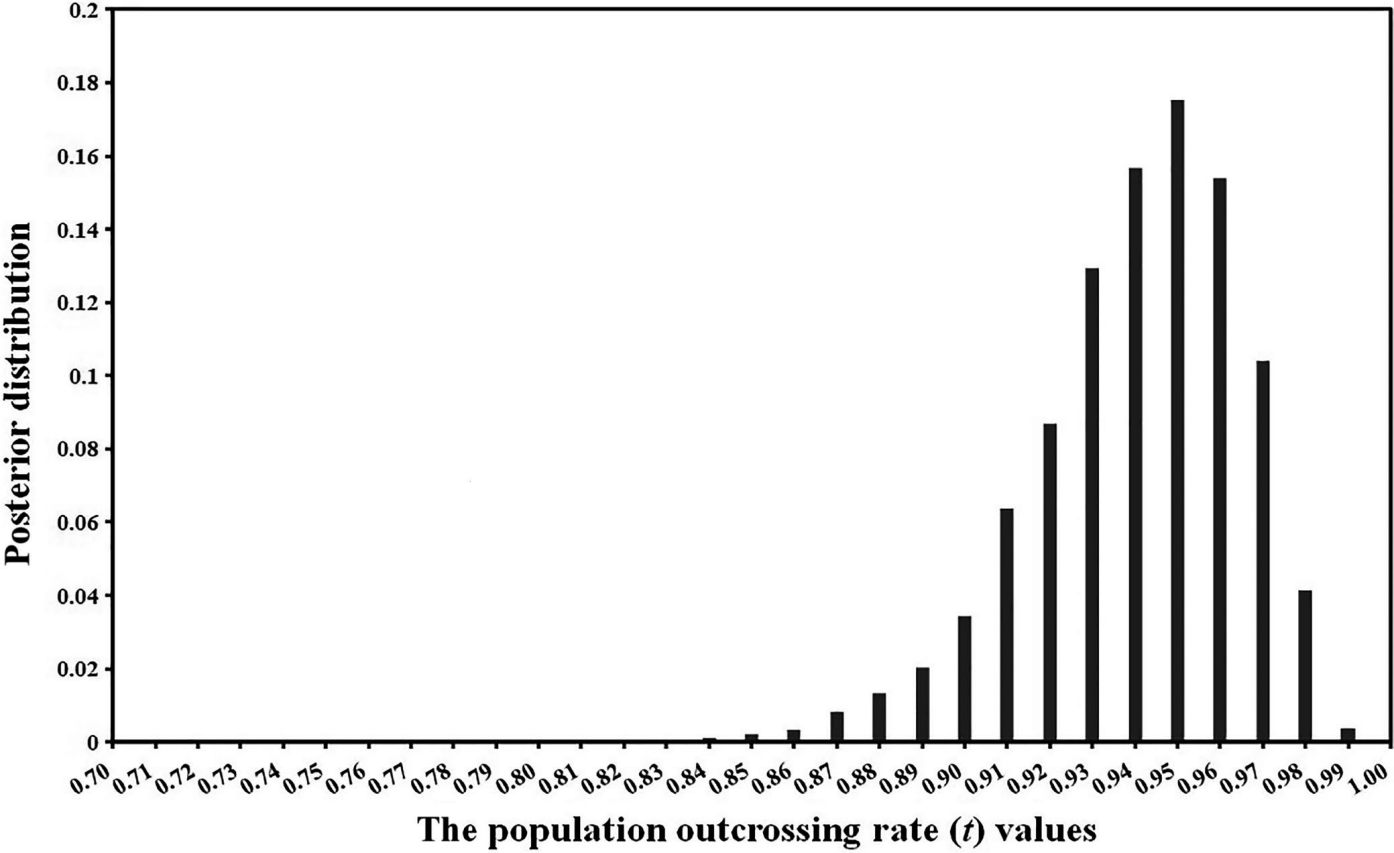
Posterior distribution of estimated *t* obtained using the BORICE. The distribution from every 10 steps in the chain (total step length was 1,100,000) following the burn‐in of 100,000 steps. For a given value of *t* on the *x*‐axis, the corresponding value on the *y*‐axis is the proportion of the chain yielding that *t* value

From microsatellite analysis, the posterior distribution of estimated *t* based on the BORICE is shown in Figure 5, and the maximum posterior *t* was 0.95 (2.5 percentile = 0.89, 97.5 percentile = 0.98). The maximum value of the posterior distribution for *F* was 0 (2.5 percentile = 0, 97.5 percentile = 0). The average ln likelihood for this model was −1,668. Examining the posterior distributions of inbreeding histories for maternal plants, we found that the most probable IH value (*C_k_*) = 0 for all individuals. At the same time, we only used three loci to evaluate the parameters of mating system. It was found that the posterior distributions of *t*, *F* and inbreeding histories of maternal individuals were basically the same, but the average ln likelihood was −624, greater than that of all loci.

The OCI was calculated by summing the following values: (a) Diameter of the flower was 6.77 ± 0.39 mm (*n* = 50) > 6 mm (Table 2), then the value was 3; (b) temporal separation between anther dehiscence and stigma receptivity: homogamy, then the value was 0; (c) spatial position of stigma and anthers: partial spatial separation (ASD = 0.59 ± 0.20 mm, *n* = 50, Table 2), then the value was 0 or 1. The OCI was 3 or 4 accordingly. According to the observation, there were 4 ovules and 4 anthers per flower in *V. negundo* var. *heterophylla*, and the P/O was 1,015 ± 218, which was the same as the number of pollen grains per anther according to the formula.

## 4 DISCUSSION

### 4.1 What is the flowering phenology of *Vitex negundo* var. *heterophylla*?

The *V. negundo* var. *heterophylla* growing in the natural environment of north China flower from June to August once a year, and fruit from July to September. A similar pattern in flowering period was also documented in other species of the genus *Vitex*: *V. rotundifolia* (Murren et al., 2014), *V. rapinii* (Kok, 2007), and *V. agnus‐castus* (Stojković et al., 2011). It is reported that the flowering period of *V. negundo* occurs during June–November or during January–May (Bhat, Jain, & Sharma, 2013), which is notably different from that in *V. negundo* var. *heterophylla*, although a slight overlap in blooming phase does exist. The primary factor contributing to this phenomenon is the difference in environmental factors in study areas such as temperature, light, and rainfall. Consequently, the variation in mating system between two sibling species stemming from different flowering phenology merits further studies.

Terminal panicles produce numerous small flowers as attractive units in 3–4 weeks, reflecting an investment in floral rewards. At certain times, the availability of abundant flowers will encourage pollinators to remain on the same plant, meeting their feeding needs and favoring self‐pollination or geitonogamy (Harder & Barrett, 1995; Pool‐Chalé et al., 2018). However, the traits of nectar of abundant flowers can be changeable between floral phases within the same inflorescence to force insects to move between individual plants, which may probably avoid geitonogamy (Antoń & Denisow, 2014; Carlson, 2007). Flowering among individuals is not synchronized, which increases the attraction to pollinators and facilitates the pollen exchange. When flowering intensity is high, the significant degree of overlap in flowering time within an inflorescence increases the possibility of geitonogamy (Nazareno & Reis, 2012; Roccotiello et al., 2009). However, when flowering intensity is low, geitonogamy is unlikely to occur due to the distance between single flowers.

Floral longevity as a key characteristic for plant reproduction balances rates of pollen transfer and dispersal against the cost of floral maintenance (Ashman & Schoen, 1994). The flowers of *V. negundo* var. *heterophylla* featured a lifespan of 1–2 days. When flowers have high fitness accrual rates and high cost of floral maintenance, they tend to have shorter longevity (Ashman & Schoen, 1996). Moreover, the prolongation of a single flowering period during rainy days is beneficial to extend the pollination time and improve the chances of pollination when pollinators are scarce, it can be regarded as a mechanism for reproductive assurance.

### 4.2 The relationship between morphological characteristics and reproductive traits

Most flowering plants are transported by animals (Ollerton, Winfree, & Tarrant, 2011). Likewise, *V. negundo* var. *heterophylla* exhibits several floral characteristics that are adapted to insect pollination: (a) The species is characterized by having zygomorphic, two‐lipped flowers. The lower lip of the bilabiate corolla is flat and broad, which can be used as a comfortable platform for insects to find food and stop (Figure 1d–g); (b) panicles gather the flowers closely, thus reducing the time for insects to fly and search for nectar or pollen, and helping insects optimize their energy for foraging; (c) flowers attract insects by secreting nectar and emitting scent (Faegri & van der Pijl, 1979). Nectar is produced at the base of corolla tube, which is long and covered by dense hairs. This serves as a preliminary screening for the species of nectar robbers. Only the insects that can carry out the behavior of intrafloral feeding are allowed as primary nectar robbers.

Although only a relatively few flowering plants exhibit ambophily (pollinated by both insects and wind) (Yamasaki & Sakai, 2013), *V. negundo* var. *heterophylla* also shows the typical characteristics of anemophilous plants: (a) The exposed anthers and stigma are not hindered by the corolla, which is conducive to spreading and receiving pollen under the effect of wind; (b) long inflorescences can be swung in the wind to facilitate pollen dispersal (Yamasaki & Sakai, 2013). A more specific outcrossing strategy needs to be verified by artificial pollination experiments.

In addition to the obvious characteristics of outcrossing, *V. negundo* var. *heterophylla* shows several mechanisms that can promote self‐fertilization. First, flowers exhibit hermaphroditic with a short ASD (0.59 ± 0.20 mm), similar to that recorded in other species of the same genus (Ahenda, 1999; Schmidt, 2000; Sinébou et al., 2016). Furthermore, pollen activity and stigma receptivity showed the same trend over time, facilitating self‐fertilization. This homogamous feature was also found on other species of the *Vitex*, such as *V. doniana* (Sinébou et al., 2016), *V. fischeri*, and *V. keniensis* (Ahenda, 1999). *V. negundo* var. *heterophylla* populations are polymorphic for spatial position of anthers and stigma, with partial herkogamy (stigma situated higher than anthers). Herkogamy can prevent autonomous selfing due to lack of physical contact between anthers and stigma. We conclude from the flower morphological characteristics mentioned above that *V. negundo* var. *heterophylla* may present a mixed mating system dominated by outcrossing. The formation of a mating system is the result of trade‐offs and compromise of multiple selective pressures. Therefore, we hypothesize that the spatial difference in position between stigma and anthers might be the product of unstable selective pressures during mating system evolution. Notably, extensive observations under additional habitats are required in the future.

*V. negundo* var. *heterophylla* flowers secrete a small amount of concentrated nectar 24 hr after flowering (0.478 ± 0.028 μl^−1^ flower^−1^ day^−1^), and the volume and sugar content of nectar produced in the late flowering stage are significantly higher than those in the early stage (*p* < .01), which may be a mechanism for increasing the attraction to pollinators and ensuring successful reproduction. The sugar concentration in nectar varied from 33% to 57%, and this range greatly increased the possibility of providing food for a variety of pollinators. According to our observations between 9:00 to 18:00, nature populations of *V. negundo* var. *heterophylla* are principally visited by bees, wasps, ants, and butterflies. This is congruent with the general assumption that nectar with high sugar concentration (regularly >35%) attracts mainly nectar‐feeding insects such as bees and wasps (Heyneman, 1983).

### 4.3 Reproductive strategies and outcrossing rate in the natural population

For the value of OCI (3 or 4), the mating system of *V. negundo* var. *heterophylla* is defined as facultative xenogamy according to the criterion set by Dafni (1992). For the value of P/O (1,015 ± 218), the mating system belonged to facultative xenogamy with self‐compatibility according to the criterion set by Cruden (1977), which was consistent with OCI evaluation. Similar mixed mating systems have been reported in other species of the same genus, such as *V. lucens* (Barrell, Richardson, & Gardner, 1997), *V. doniana* (Sinébou et al., 2016), *V. rotundifolia* (Murren et al., 2014), *V. fischeri* (Ahenda, 1999), and species of other genera. Mating systems with complete outcrossing or complete selfing are usually regarded as stable reproductive strategies, but in the absence of outcrossing during limited pollination conditions, the selection of selfing provides a certain reproductive guarantee for plants (Goodwillie, Kalisz, & Eckert, 2005; Lloyd, 1992), although this reduces the amount of genetic diversity (Takebayashi & Morrell, 2001). Therefore, the existence of a mixed mating system balances the advantages and disadvantages of selfing and outcrossing, which is a mechanism allowing the plant to adapt to environmental conditions, and improves the chances of reproductive success in unpredictable environments.

We used SSR markers to quantitatively evaluate the mating system of *V. negundo* var. *heterophylla* at the population level for the first time. The species shows a higher outcrossing rate (*t = *0.95). *F* and inbreeding histories of all maternal individuals estimated from BORICE were 0, indicating severe inbreeding depression in this species. This is congruent with the hypothesis that inbreeding depression is apparently severe in long‐lived and mixed mating species with selfed seed never survive to adulthood (Scofield & Schultz, 2006). It is worth noting that the evaluation of outcrossing rate in this study was carried out at the seedling growth stage, and the inbreeding depression in *V. negundo* var. *heterophylla* that occur at the stage of seed maturation and germination success was skipped. Therefore, the estimated outcrossing rate of the seedlings of *V. negundo* var. *heterophylla* progeny is indeed greater than the real outcrossing rate. We suggest that in order to get more realistic results, such studies should be carried out in the seed stage of progenies.

Considering the floral morphology (morphological characteristics of outcrossing and selfing are coexist), OCI, P/O, and outcrossing rate, we give it a conclusion that the mating system of *V. negundo* var. *heterophylla* belongs to the mixed mating system dominated by outcrossing. Although SSR based analysis of the mating system provides important information on the reproductive traits of *V. negundo* var. *heterophylla*, the research objective of this study involves a single population within a limited time period. Mating patterns vary greatly with population genetic structure, service level of pollinators, geographical distribution, etc (Charbonnel, Rasatavonjizay, Sellin, Bremond, & Jarne, 2005; Goodwillie et al., 2010). Consequently, it is necessary to conduct more extensive research on the reproductive strategies of *V. negundo* var. *heterophylla*.

## 5 CONCLUSION

This study provides an overview of the reproductive traits of *V. negundo* var. *heterophylla* mainly by morphological observations and SSR markers experiment. *V. negundo* var. *heterophylla* is a hermaphroditic and homogamous species, presenting a predominantly outcrossed mixed mating system with inbreeding depression. The high level of outcrossing rate in the natural population proves that this species has a wide range of reproductive sensitivity and genetic variation, which is important for evolution. These reproductive traits presented here provide scientific basis for the protection of plant resources, the effective use of slope protection function, and the breeding of wild varieties of *V. negundo* var. *heterophylla*. This study also provides a theoretical background for subsequent studies of pollination biology and population genetic structure.

## CONFLICT OF INTEREST

The authors have declared that no competing interests exist.

## AUTHOR CONTRIBUTION

**Xiaohan Sun:** Conceptualization (equal); Investigation (lead); Methodology (equal); Writing‐original draft (lead). **Feng Wang:** Investigation (supporting). **Rong Cui:** Investigation (supporting). **Xiao Liu:** Conceptualization (supporting); Methodology (supporting); Software (equal). **Xiangxiang Li:** Investigation (supporting). **Jibin Dong:** Investigation (supporting). **Lu Sun:** Investigation (supporting). **Siqi Qin:** Investigation (supporting). **Renqing Wang:** Conceptualization (equal); Methodology (equal); Writing‐review & editing (equal). **Peiming Zheng:** Conceptualization (equal); Methodology (equal); Writing‐review & editing (lead). **Hui Wang:** Conceptualization (equal); Methodology (equal); Writing‐review & editing (equal).

## Data Availability

The data that support the findings of this study are openly available in Dryad. Floral morphological data and microsatellite genotypes: Dryad https://doi.org/10.5061/dryad.qrfj6q5c5. Nectar rewards data, OCI data, and pollen viability data: Dryad https://doi.org/10.5061/dryad.qrfj6q5c5.
